# Pain and Nutrition in Dementia and Alzheimer’s Phase 1: a cross-sectional, observational study design

**DOI:** 10.3389/frdem.2026.1789761

**Published:** 2026-03-31

**Authors:** Taylor C. Judkins, Hailey J. Andrews, Qianqian Song, Edward I. Clark, Camesha Tate, Joshua I. Wais, Roger Fillingim, Zhiguang Huo, Steven T. DeKosky, Barabara Gower, Ronald A. Cohen, Natalie C. Ebner, Yenisel Cruz-Almeida, Larissa J. Strath

**Affiliations:** 1 Division of Clinical and Population Health Integration, Department of Health Outcomes and Biomedical Informatics, College of Medicine, University of Florida, Gainesville, FL, United States; 2 Department of Community Dentistry and Behavioral Science, College of Dentistry, University of Florida, Gainesville, FL, United States; 3 Pain Research and Intervention Center of Excellence (PRICE), University of Florida, Gainesville, FL, United States; 4 Claude D. Pepper Older Adults Independence Center (OAIC), University of Florida, Gainesville, FL, United States; 5 Department of Neurology, College of Medicine, University of Florida, Gainesville, FL, United States; 6 HCA Florida Ocala Hospital, Ocala, FL, United States; 7 Department of Biostatistics, College of Public Health and Health Professions, University of Florida, Gainesville, FL, United States; 8 Boehringer Ingelheim, Ridgefield, CT, United States; 9 Center for Cognitive Aging and Memory, University of Florida, Gainesville, FL, United States; 10 Evelyn F. and William L. McKnight Brain Institute, University of Florida, Gainesville, FL, United States; 11 Department of Nutrition Sciences, School of Health Professions, The University of Alabama at Birmingham, Birmingham, AL, United States; 12 Department of Clinical and Health Psychology, College of Public Health and Health Professions, University of Florida, Gainesville, FL, United States; 13 Department of Psychology, College of Liberal Arts and Sciences, University of Florida, Gainesville, FL, United States; 14 Department of Neuroscience, College of Medicine, University of Florida, Gainesville, FL, United States

**Keywords:** Alzheimer’s disease, cognition, dietary inflammatory index, epigenetic aging, high impact pain, neuroinflammation, osteoarthritis

## Abstract

**Background:**

Neurodegenerative diseases such as Alzheimer’s Disease and related dementias (ADRDs) as well as chronic pain have increased in prevalence as the population ages. In fact, recent epidemiological research suggests that having chronic pain may increase one’s risk of all-cause dementia. There are mechanistic factors that overlap in both ADRD and chronic pain progression, including epigenetic dysregulation that could lead to increased inflammation. Previously, our group presented evidence that dietary patterns impact inflammatory potential and epigenetic modifications, and accelerate epigenetic aging. Here, we hypothesize that diet- induced inflammation and epigenetic alterations may be underexplored mechanistic pathways connecting chronic pain and ADRD risk.

**Methods:**

The Pain and Nutrition in Dementia and Alzheimer’s Phase 1 (PANDA-1) study is a cross-sectional, observational study, which will recruit 90 individuals ≥55 years of age with and without painful knee osteoarthritis (OA). Biological samples will be collected to assess study eligibility, blood-based inflammatory markers, and epigenetic age using the epigenetic clock DNAmGrimAge. A 24-h dietary recall will be completed to determine nutrition status via the Dietary Inflammation Index (DII). Pain and psychosocial questionnaires will be employed to determine pain phenotypes. Quantitative Sensory Testing will be conducted to determine responses to noxious mechanical and thermal stimuli. Lower-extremity function and mobility measures will also be obtained. Finally, height, weight, pain history, medical history, medication use, and demographic variables will be collected as covariates. Hierarchical regression, mediation and moderation analyses, as well as ANOVAs will be conducted to evaluate relationships among the DII, epigenetic aging, cognition status, and pain.

**Conclusion:**

This study will integrate dietary, epigenetic, and cognitive assessments in a chronic pain population, to lay the groundwork of a possible associations linking chronic pain and ADRDs. PANDA-1 aims to determine potential relationships of dietary patterns on interindividual variability of cognitive status and pain outcomes in older adults deemed cognitively intact. Subsequent phases of this study will include individuals with mild cognitive impairment and ADRDs. Findings from this work will inform future studies targeting dietary intervention approaches to mitigate overlapping neurodegenerative and pain-related aging processes.

## Background

Chronic pain is highly prevalent starting in middle age and prevalence increases as individuals grow older (Domenichiello and Ramsden, 2019). Globally, approximately 1.5 billion people experience chronic pain; in the United States (US) alone, new estimates suggest that 1 in 5 individuals will have chronic pain at any given time, leading to significantly negative physical, psychosocial, and economic costs (Yong et al., 2022). In the US, chronic pain represents a major public health issue, as it is the most common reason for seeking medical care, increases the risk of physical and cognitive disability, and contributes to increased risk of opioid abuse (Roos et al., 2008). Since chronic pain is more prevalent in older adults and the number of adults over the age of 65 is expected to increase from 58 to 82 million by 2050 in the US alone, the health concerns and expenses related to chronic pain for both older individuals and society at large will increase significantly in the coming decades (Cire, 2016).

Like chronic pain, the number of individuals living with Alzheimer’s Disease and Related Dementias (ADRDs) in the US is expected to almost triple to 14 million in the next 30–40 years (GBD 2019 Dementia Forecasting Collaborators, 2022). ADRD symptoms vary at the early stages of the disease and increase in severity as the disease progresses. These symptoms include cognitive, behavioral, psychological, and physical symptoms, such as memory loss, inability to communicate, mood swings, falling, jumbled speech, and the inability to coordinate movement (Bature et al., 2017). Due to the rapidly increasing older population, health concerns at the patient and healthcare system levels related to ADRDs will also significantly increase, especially if research distinguishing etiologies, successful interventions, and effective prevention strategies are not accomplished.

The literature supports a bidirectional association between chronic pain and ADRDs, though a clear mechanistic link between these two conditions remains to be elucidated. Almost half of ADRD patients report having chronic pain, and pain intensity is also positively correlated with dementia severity (Yao et al., 2025). During the preclinical phase of ADRDs, there is a 20- to 30-year span where cognitive deficits may not yet be present, but systemic, pathological changes may already be occurring (Seifan et al., 2019). Interestingly, it is during this prodromal age range (~45 + years) prior to ADRD diagnosis that we begin to see the peak in diagnoses of multiple chronic pain conditions (Rustøen et al., 2005). This prodromal period also is marked by alterations in inflammatory signaling that are also implicated in chronic pain conditions. Nervous and immune system dysfunction and chronic inflammation are now also recognized as key factors in ADRDs leading to neurodegeneration and cognitive decline as well as chronic pain (Bayraktaroglu et al., 2025; Totsch and Sorge, 2017). In addition, recent reports suggest that chronic widespread pain predicts cognitive decline, as chronic pain is associated with a 43% increase in all-cause dementia, and a 47% increase in ADRD risk (Wang et al., 2024; Li et al., 2024; Bornier et al., 2023; Innes and Sambamoorthi, 2020).

Although epidemiological studies point to a relationship between these two conditions, there are still major gaps in the literature that must be filled in order to truly understand the connection between chronic pain and ADRDs. Of note, the majority of the current literature is epidemiological in nature, and backwards translation to clinical and basic science methods must be completed to determine clinically relevant factors and underlying mechanisms. Additionally, the consideration of pain impact (i.e., high or low) on cognition, a proxy that includes not only pain severity but also captures its influence on quality of life and related disability, has so far been overlooked. In fact, high-impact chronic pain is a subcategory of chronic pain that significantly impacts daily life and functioning, such as work, socializing, and self-care. Individuals affected also appear to have a greater magnitude of association with a variety of comorbidities such as depression, anxiety, sleep disturbances, immune and inflammatory dysfunction, and cardiometabolic diseases (Zelaya et al., 2020; Fang et al., 2023). Moreover, studies implicating lifestyle factors such as nutrition as possible mechanism influencing the link between chronic pain and ADRD have not been completed yet; despite evidence of nutrition status influences on both conditions independently (Strath et al., 2021; Strath et al., 2022a; Strath et al., 2022c; Grant and Blake, 2023; Xu Lou et al., 2023). Dietary patterns are known to modulate immune function and systemic inflammation, suggesting a plausible pathway through which nutrition may simultaneously influence pain, accelerated biological aging, and cognitive health. Finally, epigenetic modifications through one’s diet pattern that could contribute to immune system dysfunction and ADRD and chronic pain outcomes, such as DNA methylation and epigenetic/cellular aging, has not been appreciated (García-García et al., 2024).

**Figure 1:**
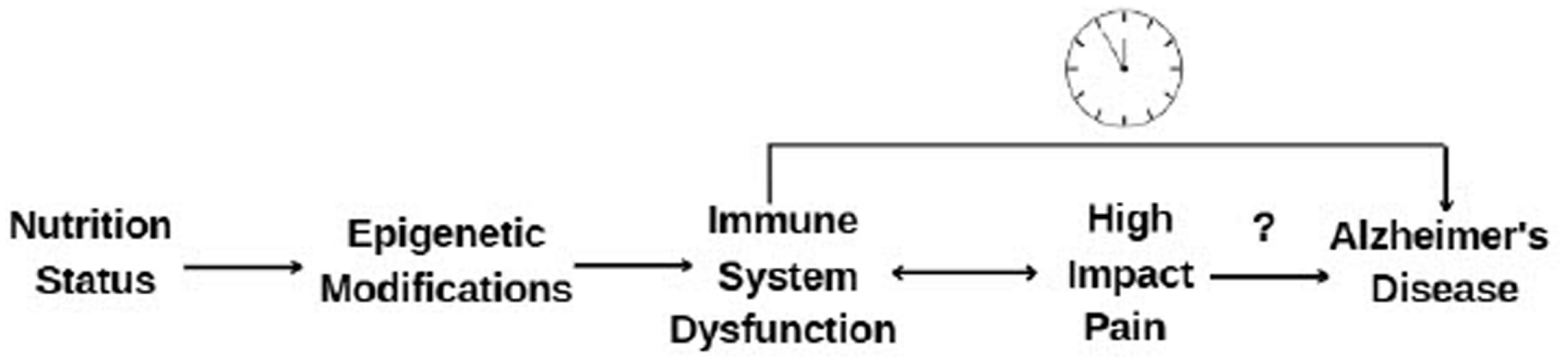
Hypothesized mechanistic model of the relationship between nutrition, high impact chronic pain, and Alzheimer’s disease and related dementias.

To begin to fill these gaps, the aim of this observational study is to determine associations between diet patterns captured via the Dietary Inflammatory Index (DII), epigenetic clocks and cellular aging, on cognitive function in individuals across the middle-age to older adults 55 years of age or older with varying levels of pain (low, high, pain-free controls; see conceptual model in Figure 1). We hypothesize that individuals with chronic pain will (A) have a more positive/inflammatory DII, which will be associated with (B) accelerated epigenetic aging and (C) lower scores on cognitive exams. We further predict that (D) these associations will be stronger in the high-impact than the low-impact pain and pain-free controls.

## Methods

**Figure 2:**
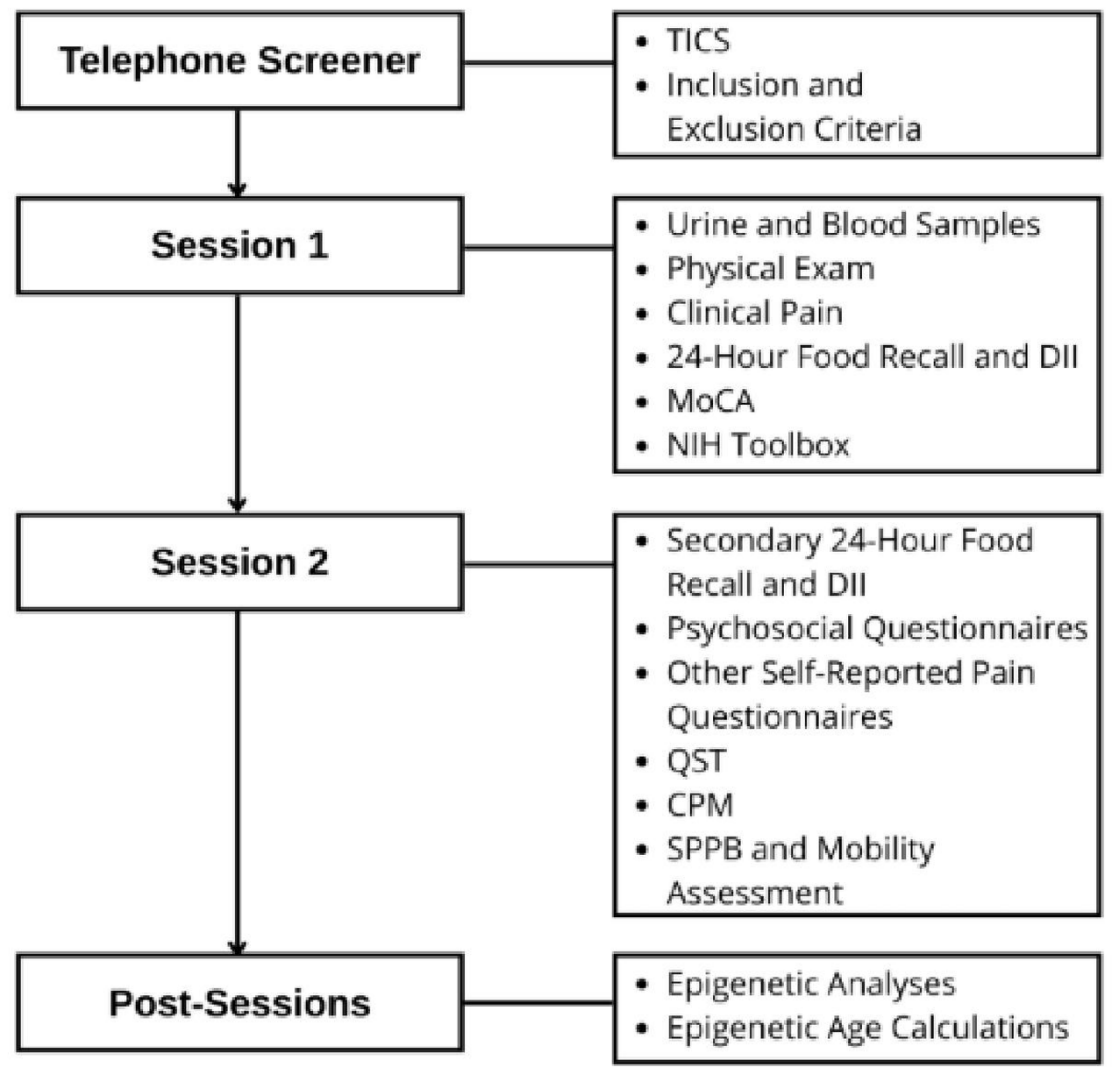
Study design.

We propose a cross-sectional, observational study that will assess the relationships among dietary patterns, chronic pain, cognition, and epigenetic differences in clinically assessed, cognitively intact older adults with and without symptomatic knee osteoarthritis (KOA) pain (Figure 2). To reduce participant burden, data collection will be completed in 2 experimental sessions approximately 1 week apart. In short, after completing initial screening, eligible participants (*n* = 90) will undergo the health and sensory assessments for collection of clinical (pain) and functional (physical, cognitive, emotional) status, undergo quantitative sensory testing (QST), and provide samples for blood-based biomarker data.

### Study participants

This study is ancillary to the Understanding Cognition, Oxytocin, and Pain in Elders Study (UCOPE; R01AG059809; NCT03878589). The primary focus of the parent grant was to assess effects of intranasal oxytocin (vs. a placebo) on pain outcomes via a double-blind, cross-over, randomized control trial among middle-aged and older adults. Only methods relevant to the present PANDA study will be detailed below. Full study methods of the UCOPE clinical trial can be found elsewhere (Cruz-Almeida et al., 2025). Data will be collected from 90 adults 55 years and older with and without painful KOA at two of the baseline sessions prior to the first arm intervention. This age range was selected to capture individuals within middle-to-older adulthood when chronic pain prevalence and early neurodegenerative changes increase (Domenichiello and Ramsden, 2019; Singh et al., 2023; Cheng et al., 2025). For individuals with painful knee OA, eligibility criteria include older adults over 55 years of age who meet American College of Rheumatology clinical criteria for KOA: pain of at least 6 months duration, pain experienced on more than 4 days/week, and with moderate pain at baseline (i.e., > 3/6 on a Visual Descriptor Scale), will be considered for participation (Altman et al., 1986). For those without pain, eligibility includes not having chronic KOA pain and meeting the remaining inclusion/exclusion criteria described below. For this phase of the PANDA study, only clinically assessed, cognitively intact older adults, defined by a person’s cognitive status (e.g., thinking, learning, reasoning, and memory) will be included. We will examine interindividual differences in cognitive functioning among individuals with and without chronic KOA pain, and small, but possibly relevant declines in individuals with pain compared to those without. In future iterations of the PANDA study, we plan to include individuals with subjective cognitive impairment, mild cognitive impairment, and ADRDs to assess these relationships across the disease course. Both community-based (i.e., flyers, town halls, mailers) and clinic-based recruitment methods will be utilized. Additionally, University of Florida Institutional Review Board (IRB) approved registries will be used to contact potential participants.

Participants will be excluded if they have concurrent medical or arthritic conditions that could confound symptomatic KOA-related outcomes, or coexisting disease that could preclude successful completion of the protocol will also be excluded, including a systemic rheumatic condition (e.g., rheumatoid arthritis, systemic lupus erythematosus, or fibromyalgia), a history of clinically significant surgery to the index knee (i.e., total knee arthroplasty), uncontrolled hypertension (>150/95), poorly controlled diabetes (HbA1c > 7%), cardiovascular or peripheral arterial disease, a serious psychiatric disorder requiring hospitalization within the past 12 months or characterized by active suicidal ideation, and diminished cognitive function that would interfere with completion of study procedures (i.e., Telephone Interview for Cognitive Status (TICS)_score < 30 (see below) and/or a diagnosis of mild cognitive impairment or ADRDs). Pregnant and breastfeeding individuals will be excluded as well as individuals who are enrolled in parallel in another interventional research study (Cruz-Almeida et al., 2025).

### Sample size calculation

Based on power-analytic considerations with a power of 0.80 at an alpha level of 0.05, a total of 90 individuals (with approximately half male, half female; representative of the local community sociodemographic status) will provide sufficient power to detect a large effect size (Cohen’s d ≥ 0.8) in analyses that consider pain outcomes, cognitive outcomes, the diet pattern predictor variable, and relevant covariates described below. To account for a 20% attrition rate, a total of 113 individuals will need to be recruited. The sample size was powered to detect large effect sizes based on previous work examining epigenetic aging and pain outcomes. Analyses involving mediation and moderation will be considered exploratory due to the increased power requirements of such models.

### Initial screening

After receiving telephone consent, all potential participants will undergo an initial screening interview This screening procedure will include a KOA screening interview that has shown 87% specificity and 92% sensitivity for detecting KOA pain severity sufficient for the study (Roux et al., 2008). The initial screening will also collect demographic variables, such as age, sex, gender, race, ethnicity, health history information required for inclusion/exclusion assessment, education level, household size and employment. The TICS will be given to participants to assess cognitive status (Brandt, 2020). Eligible individuals will be scheduled for the Health Assessment Session.

### Session 1: health assessment

#### Urine and blood samples

All sessions will be performed in the morning to control for circadian effects and to reduce participant burden by providing a fasted blood sample. After written informed consent is obtained, participants will provide a urine sample to determine osmolality. Participants will then have their blood taken from a forearm vein by a trained phlebotomist. Some of the blood samples will be stored for epigenetic and nutrient analyses, and some will be used to immediately evaluate standard clinical parameters on a Comprehensive Metabolic Panel (CMP-14). Results will be reviewed by study staff and the study physician to determine study continuation. If the CMP-14 shows variables that are extremely out of range, or demonstrate evidence of diabetes, the participant will be unenrolled and encouraged to take the results to their personal physician for further evaluation. For women under the age of 63 who have not undergone total hysterectomy, pregnancy tests will be conducted at study commencement. Individuals who have positive pregnancy tests will be alerted and unenrolled from the study.

#### Physical examination

Height (cm) and weight (kg) will be measured and Body Mass Index (BMI) calculated as it is significantly related to pain symptoms in OA (Tukker et al., 2009). Participants will complete a thorough pain and medical history interview, including a review of bodily systems, as well as assessing the reported duration of KOA pain, current and past treatments for KOA pain, other painful sites and their duration, comorbid conditions that were not exclusionary, hospital admissions in the past 3 years and current medication and dietary supplement use as in our previous studies (Strath et al., 2022a; Strath et al., 2022c; Strath et al., 2024; Strath et al., 2022b; Overstreet et al., 2022). Symptomatic hip OA is common in the KOA population and is associated with KOA pain and disability (Wood et al., 2008; Dawson et al., 2004; Cecchi et al., 2008). As such, it will be included as a covariate, and details regarding the presence of this condition will be captured in the pain and medical history interview. Information regarding hormone replacement therapy will be obtained for both sexes. For females assigned at birth, information regarding menopausal status (naturally occurring with time, or induced due to total hysterectomy/oophorectomy) will be recorded, as the shift in hormones has been shown to influence pain and cognitive outcomes after menopause (Ebner et al., 2014; Strand et al., 2025). If there are concerns with eligibility, participants will undergo a second physical examination by the study physician to confirm the diagnosis of KOA according to American College of Rheumatology criteria noted above.

#### Clinical pain

Clinical pain will be assessed according to location, duration, overall pain severity, and pain interference with daily activities. These measurements will encompass the key dimensions for pain assessment as recommended by the American Geriatrics Society and according to the pain taxonomy by the International Association for the Study of Pain (Wood et al., 2008; Raja et al., 2020). A Verbal Descriptor Scale (VDS) will assess changes in pain intensity before and after treatment. The VDS ranges from “No pain” (scored as 0) to “The most intense pain imaginable” (scored as 6). This scale is considered easier to understand and preferred over numerical rating scales by older individuals (Ware et al., 2006). The Western Ontario and McMaster Universities Osteoarthritis Index (WOMAC), a reliable, well-validated measure of lower extremity pain and function in individuals with knee OA, will also be administered (Bellamy et al., 1988). Summary scores of the WOMAC range from 0 (No pain or disability) to 96 (Extreme pain or disability). Finally, the Brief Pain Inventory – Short Form (BPI-SF) will also be administered to assess pain severity and related disability, and will also be used to determine pain impact grouping (Tan et al., 2004). The BPI-SF is a 9-item, widely used questionnaire used in both research and clinical practice to assess pain severity over time and to assess the degree to which a person’s pain may interfere with daily life in activities such as walking, work, homelife, mood and sleep. Pain severity is calculated as the mean of 4 items (worst, least, average, and current pain), while pain interference is calculated as the mean of 7 items assessing interference with daily functioning. Consistent with prior definitions of high-impact chronic pain, participants will be categorized as either high-impact pain (BPI interference ≥5), low-impact pain (BPI interference <5), or pain-free controls (absence of chronic knee pain) (Dahlhamer et al., 2018).

#### Dietary inflammatory index (DII)

The DII was developed to provide a quantitative means of assessing the role of diet in relation to health outcomes ranging from blood concentrations of inflammatory cytokines to other chronic diseases. Diet data will be collected via a 24-h food recall during the first session and will be analyzed using the Automated USDA Multiple Pass Method (Steinfeldt et al., 2013). In short, participants will be asked to document food and beverages consumed, as well as supplements taken, 24 h prior to their visit. Assessments will be conducted in an interview by a trained research assistant to probe deeper into the details of meals, asking for variables such as (but not limited to) cooking methods, salt and spices used, or the presence or absence of dipping sauces for meals or ice in beverages. Visual aids such as standard measuring cups, plates, and drink cups in various sizes will be provided to aid in accuracy.

These data will then be entered into the Nutrition Data System for Research (University of Minnesota) for nutrient analysis by trained research assistants, the Principal Investigator, and a Registered Dietician. Data will be entered a minimum of 2 times and compared for interrater reliability. Following quantification, nutrient values will be used to calculate DII score according to previously published methods that have been translated into Python code^1^ (Shivappa et al., 2014). Approximately 28–30 dietary components available in the NDSR dataset will be included in the DII calculation. For components not captured in the dietary recall system, values will be treated as missing and excluded from the standardized z-score calculation according to published DII methodology. Energy-adjusted DII (E-DII) scores will also be computed to account for total caloric intake. Although DII is the *a priori* independent variable, additional analyses may be conducted using other diet components as suggested by the literature for their potential involvement in pain and cognition, as the NDSR output is extensive and can provide this opportunity. It is important to note that although two recalls are widely used in epidemiologic research, they may not fully capture habitual dietary intake.

#### Montreal cognitive assessment

The Montreal Cognitive Assessment (MoCA) is a brief, standardized screening instrument designed to detect mild cognitive impairment and early cognitive changes that may not be captured by other short cognitive tests (Freitas et al., 2013). The MoCA provides a comprehensive evaluation across multiple cognitive domains on a 30-point scale. It assesses visuospatial and executive functioning through tasks such as trail-making, clock drawing, and cube copying; naming abilities via identification of low-frequency animal images; and memory through delayed recall of a five-word list. Attention and working memory are evaluated using digit span, sustained attention tasks, and serial subtraction, while language skills are assessed through sentence repetition and phonemic verbal fluency. The test also measures abstraction by asking individuals to identify similarities between paired words, and orientation through questions regarding time and place. Collectively, these components allow the MoCA to serve as a sensitive tool for identifying subtle cognitive deficits in clinical and research settings. Presently, to ensure that this cohort is cognitively intact, a cutoff score of 26 out of 30 points will be used in case the TICS does not adequately capture their exclusionary impairment (Sun et al., 2023). If a participant does not meet this criteria, they will be removed from the remainder of the study.

#### National Institutes of Health toolbox

The National Institutes of Health (NIH) Toolbox Cognitive Assessment is a standardized, computerized battery designed to measure a broad range of cognitive functions across the lifespan, from childhood through older adulthood (Rose et al., 2025). It was developed to provide efficient, reliable, and comparable cognitive data for epidemiologic studies and clinical research, the battery includes tests that evaluate key cognitive domains such as executive function, attention, episodic memory, processing speed, and language. Core measures included in the PANDA study were tasks assessing inhibitory control and cognitive flexibility (Flanker Inhibitory Control and Attention Test; Dimensional Change Card Sort), working memory (List Sorting Working Memory Test), episodic memory (Picture Sequence Memory Test), processing speed (Pattern Comparison Processing Speed Test), and vocabulary knowledge (Picture Vocabulary Test; Oral Reading Recognition Test). Each test uses adaptive or performance-based formats to ensure sensitivity across a wide range of abilities.

### Session 2: sensory assessment visit

#### Second 24-h food recall and DII calculation

To capture what the participant is eating on average, a second 24-h food recall will be conducted during the second session following the same protocol detailed above. The DII will also be calculated in the same fashion. The mean of the DII scores from the two sessions will be used as the DII variable in the analyses.

#### Psychosocial questionnaires

Multiple psychosocial factors have been related to chronic pain, cognitive status and diet patterns and must always be included as covariates (Tanner et al., 2025). Thus, we will assess psychosocial factors across the following domains:

##### Pain coping

The Coping Strategies Questionnaire-Revised (CSQ-R) consists of 27 items relating to how individuals cope with pain (Riley et al., 1999). It comprises six subscales based on pain coping strategies that individuals report (i.e., diverting attention, catastrophizing, praying and hoping, ignoring pain sensations, reinterpreting pain sensations, and coping self-statements).

##### Affective distress

We will use two validated measures to assess this domain. The Beck Depression Inventory, 2nd Edition (BDI) is a widely used depression scale that assesses affective (e.g., sadness, loss of interest), cognitive (e.g., worthlessness, guilty feelings), and somatic (e.g., changes in sleep, tiredness or fatigue) symptoms common among depressed individuals (Steer et al., 2000). It contains 21 self-report items assessing the frequency and severity of depressive symptoms over the previous 2 weeks. The Positive and Negative Affect Scale (PANAS) is a 20-item scale that assesses positive and negative affect (Watson et al., 1988). For this study, participants will be requested to provide “state” information by responding to items “at the present moment.”

##### Life satisfaction/quality of life

We will use two validated measures to assess these domains. We will assess self-reported quality of life using the 36-Item SF Survey Quality of Life, which assesses 8 domains including physical functioning, role limitations as a result of physical problems, bodily pain, general health perception, vitality, social functioning, role limitations resulting from emotional problems, and general mental health (Brazier et al., 1992). The Satisfaction with Life Scale (SWLS) is a short 5-item questionnaire that will assess satisfaction with people’s lives as a whole (Diener et al., 1985).

##### Stress

As above, we will use two validated measures to collect data related to the participant’s stress. The Perceived Stress Scale – 14 Item (PSS-14) is a well validated psychological tool that measures how unpredictable, uncontrollable and overloaded individuals find their lives, assessing the degree to which situations were appraised as stressful over the past month (Lee, 2012). The Daily Stress Inventory (DSI) is a self-reported instrument designed to measure the frequency and intensity of minor, everyday stressful events experienced in a 24-h period (Brantley et al., 1987).

#### Other self-reported pain questionnaires

To complement the qualitative pain history and clinical pain measures captured in the first session, the following questionnaires will be delivered to assess multidimensional pain experience. Individuals will complete the PainDETECT questionnaire to assess neuropathic pain detection. The PainDETECT was developed and validated to assess this type of pain, and its components, by assessing specific sensory experiences and symptoms (i.e., burning, shocking) and pain characteristics (i.e., radiating, time-course) (Freynhagen et al., 2016). If an individual scores greater than 12, their experimental pain for each area indicated will be assessed further during the QST battery. As back pain is also a common comorbidity with KOA, we will comprehensively assess such pain using the Oswestry Low Back Pain Disability Questionnaire (Deyo et al., 2014; Fairbank and Pynsent, 2000).

#### Quantitative sensory testing

**Figure 3:**
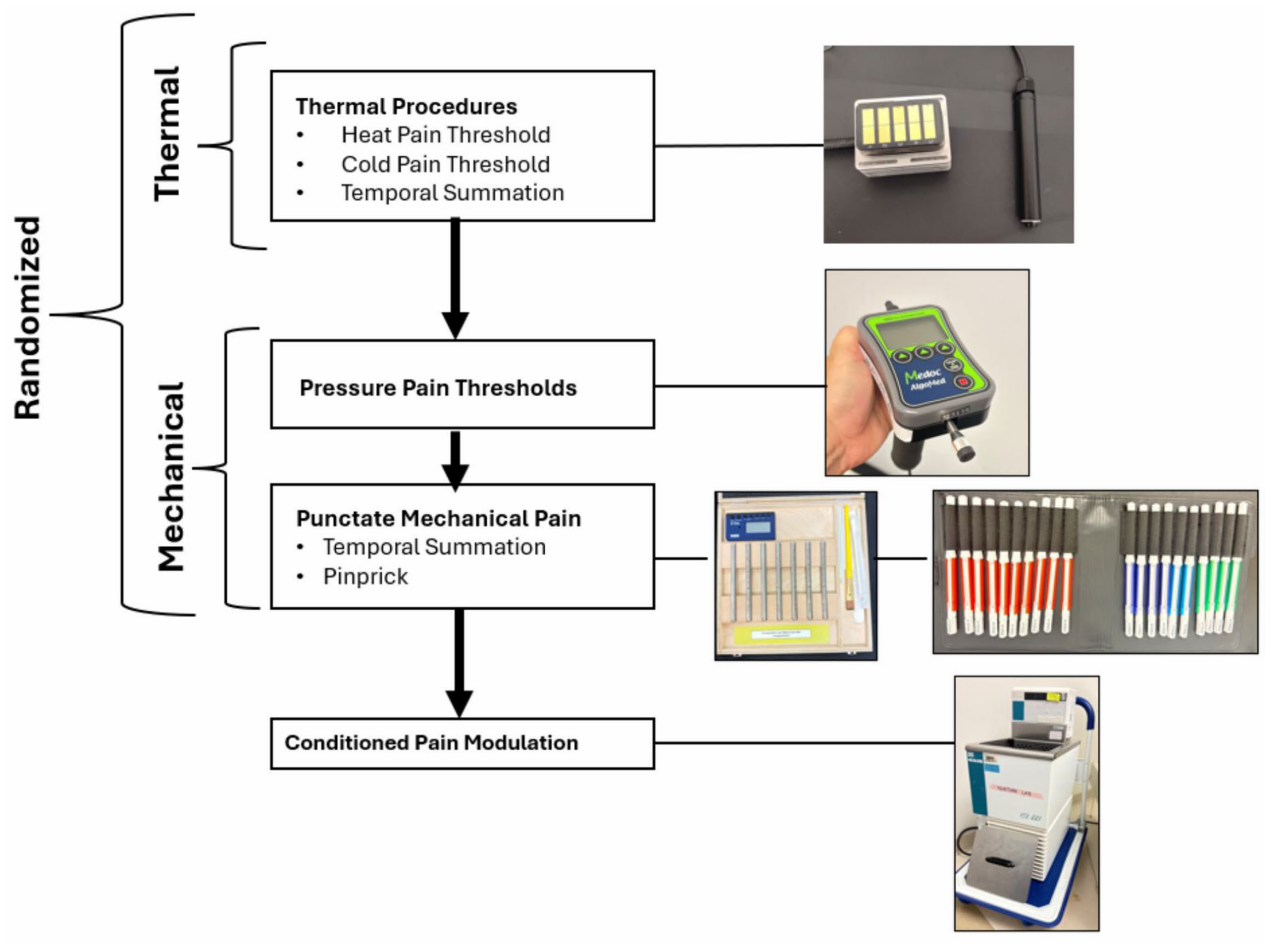
Quantitative sensory testing scheme.

Participants will undergo QST to determine responses to mechanical stimuli, thermal stimuli, and conditioned pain modulation (CPM) (Figure 3). Our group has completed QST batteries similar to the methods outlined below and are the standard of practice in pain research (Strath et al., 2022c; Cardoso et al., 2016; Cruz-Almeida et al., 2013; Strath et al., 2020). We will randomize the order of thermal and mechanical testing to control for possible peripheral and central sensitization throughout the procedures. CPM using the cold pressor will always occur last to avoid carryover effects. Patients’ medications taken that day and current clinical pain will also be confirmed prior to starting QST procedures.

#### Mechanical testing procedures

Pressure pain threshold (PPT) will be assessed at the medial and lateral joint lines of the index knee, and at the ipsilateral quadriceps and trapezius muscles. For all PPT measurements, after an initial practice trial, three trials will be conducted at each site, and their average PPT will be computed for data analysis. Using a digital handheld clinical grade pressure algometer (Algomed, Medoc, Ramat Yishai, Israel), the examiner will apply a constant rate (30 kPa/s) of pressure, and the participant will press a response button when the sensation first becomes painful, at which time the device records the pressure to be used as the PPT.

Punctate Mechanical Pain (PMP) will be assessed using the PinPrick (MRC Systems, Heidelburg, Germany). Starting at the heaviest force of 512 mN, participants will be asked to rate their pain following one contact. Contacts will then occur in a descending fashion by force, obtaining pain ratings at each level, until the last pinprick of 8 mN is reached. A second trial will be done in an ascending fashion, beginning at 8mN and ending at 512 mN, with pain ratings obtained for each. Pain ratings will be averaged by force to be used as PMP variables in analyses.

Temporal summation of mechanical pain (TSMP) will be assessed at the patella of the index knee and the dorsal aspect of the ipsilateral hand using a nylon monofilament (Touchtest Sensory Evaluator 6.65) calibrated to bend at 300 g of pressure. As in our previous studies, participants will provide a pain rating following a single contact, after which they will provide another pain rating following a series of 10 contacts at a rate of one contact per second. The difference between the pain rating for the single versus 10 contacts reflects the TSMP.

#### Thermal testing procedures

Thermal stimuli will be delivered to the most affected knee and/or to the ipsilateral forearm and/or thenar eminence, in randomized order, using a QST. Lab TCS II Thermal Stimulator (QST. Lab, Stratsbourg, France). Heat pain threshold (HPT) and cold pain threshold (CPT) will be assessed at each site. HPTs will be assessed by applying the thermode and allowing it to increase at a rate of 1 degree C/s until the participant indicates on the response clicker that their HPT has been reached. For safety, an automatic shutoff parameter will be in effect. Should the participant reach 52 °C without indicating a response, the unit will automatically shut off to prevent the skin from burning. CPTs will be assessed by applying the thermode and allowing it to decrease at a rate of 1 °C /second until the participant indicates on the response clicker that their CPT has been reached. Again, for safety, an automatic shutoff parameter will be in effect. Should the participant reach 0 °C without indicating a response, the unit will automatically shut off to protect the skin from frostbite.

Temporal summation of thermal pain (TSTP) will be assessed using a 30 × 30 mm thermode will be placed on the ipsilateral forearm at a baseline of 38 °C and allowed to rise to 45 °C (0.4 s rise time, 0.8 s peak time, and 0.4 s return to baseline of 38 °C with an onset to onset of 3.3 s) for a series of 5 heat pulses. The participants will provide pain ratings for each of the 5 pulses. The difference between the pain rating for the first stimulus versus the fifth stimulus reflects the TSTP.

#### Conditioned pain modulation

CPM will be used to assess pain-inhibitory function. The conditioning stimulus will be the cold pressor task applied to the left hand, which will be tailored for each participant to achieve a stimulus that produces moderate pain (i.e., a rating of 40–60 on the 0–100 scale) and can be tolerated for a 60-s period. The test stimulus will be heat pain applied to the opposite ventral forearm, at a stimulus intensity which produces moderate but tolerable pain. First, baseline heat pain responses will be assessed, after which the participant will immerse their hand in the cold water bath for 60 s. Immediately afterwards the heat pain will again be applied to the opposite arm and pain ratings will be obtained.

#### Short physical performance battery (SPPB) and mobility assessment

As a performance-based indicator of physical function we will use this brief assessment, consisting of four lower-extremity function measures: standing, balance, walking speed, and ability to rise from a chair. These measures have been standardized and are widely used in older populations (Cruz-Almeida et al., 2017; Cruz-Almeida et al., 2014). Balance is assessed through progressively challenging stances (side-by-side, semi-tandem, and tandem), gait speed is measured by timing a 4-meter distance walk at usual pace for the participant, and lower-body strength is evaluated by timing how quickly an individual can rise from a chair five times without using their arms. Each component of the SPPB is scored on a scale from 0 to 4, with higher scores indicating better performance, yielding a total score ranging from 0 to 12. We will also measure movement-evoked pain during each task, which is associated with central pain processing in chronic pain conditions such as KOA (Cruz-Almeida et al., 2017). During each task, participants will be asked to rate their pain from 0–100 prior to commencing the movement, halfway through the task, and immediately following task completion.

### Post-sessions: epigenetic analyses and epigenetic age calculations

Epigenetic data will be collected from the previously stored blood samples from the UCOPE trial and any additional participants recruited specifically for PANDA-1. All samples will be assayed at the end of the study to control for human error and differences in reagents used. Details regarding the protocol for processing and analyzing epigenetic data have been previously described (Montesino-Goicolea et al., 2020). In short, raw data generated by the Illumina EPIC array (.idat files) will undergo quality control and normalization prior to the calculation of differentially methylated probes (DMPs), to be used for individual gene assessment and biological pathway enrichment analyses. To calculate epigenetic age, we will use DNAmGrimAge through an online calculator.^2^ The age-adjusted AgeAccelGrim variable will be calculated as the difference between chronological age and DNAmGrimAge and used throughout the analyses. We have chosen to use this clock as it has repeatedly been the only one associated with pain outcomes in our previous work (Strath et al., 2024; Strath et al., 2022b; Tamargo et al., 2024; Peterson et al., 2022).

Epigenetic aging was quantified using DunedinPACE (Pace of Aging) and DNAmGrimAge, two validated DNA methylation-based measure that captures the rate of biological aging (Lu et al., 2019; Belsky et al., 2022). DunedinPACE and DNAmGrimAge will be calculated from normalized EPIC methylation data following published methods and treated as a continuous outcome variable in all analyses. For DunedinPACE, higher values indicate a faster pace of biological aging. For the DNAmGrimAge, the difference between the calculated DNAmGrimAge and chronological age will create the usable variable, AgeAccelGrim. These clocks were selected based on its sensitivity to systemic physiological dysregulation and its relevance to chronic pain, inflammation, and cognitive aging processes.

### Hypotheses and data-analytic strategy

It is important to note that all analyses in this study are exploratory, as this study is the first of its kind to integrate pain, diet and cognitive outcomes into one study/model and given the sample size. Prior to the testing of proposed hypotheses, each variable will be carefully examined to identify missing values, statistical outliers, and violations of relevant assumptions (i.e., Durbin-Watson, Shapiro–Wilk). Sensitivity analyses will then be completed to examine differences in results between variables with missing data and those same variables with data imputation. A corrected threshold will be set to 0.05, two-tailed. Descriptive statistics will be computed and represented as percentages or means (standard deviations) to quantify demographic characteristics, mean self-reported pain, depression, anxiety, comorbidity, and socioeconomic status values (via questionnaires). Group differences (pain impact groups) among potential covariates of interest (age, sex, race, hip-to-waist ratio, depression, anxiety, stress) as well as primary outcome variables (self-reported pain via questionnaires, MEP, QST, DII, MoCA, NIH Toolbox) will be examined using a series of one-way analysis of variance tests (ANOVAs) with follow-up/*post hoc* Bonferroni corrections. Regression models will also be constructed (linear, logistic, as well as mediation and moderation analyses) with nutrition status (DII) as the predictor/independent variable, pain/cognitive outcomes as the dependent variables (self-reported pain, MEP, QST, MoCA, NIH Toolbox), and epigenetic age either as the mediating variable. All statistical analyses employed in this study will be performed using SPSS (IBM) version 29.0 (IBM; Armonk, NY), R Studio, and/or Python.

#### Specific analyses to *a priori* hypotheses

Sequential hierarchical multiple regression models will be employed to investigate the extent to which the continuous DII variable is associated with (A) greater severity, duration, and frequency of experimental and clinical pain outcomes (self-reported pain, MEP, QST); (B) accelerated epigenetic aging; and (C) worse cognitive decline (MoCA, NIH Toolbox) between individuals with and without pain. ANOVAs will be performed to (D) examine differences in variables of interest between groups: high-impact chronic pain, low-impact pain, and pain-free controls. Additionally, three separate sequential hierarchical regression models within each group will be used to examine the extent to which higher scores DII scores are associated with epigenetic aging and cognitive outcomes (MoCA, NIH Toolbox). Variables of interest (DII, epigenetic age, cognitive status) will be entered into step 1 of the analysis, relevant clinical covariates (e.g., BMI, smoking) will be entered in step 2 of the hierarchical regression models, and relevant demographic variables (i.e., age, race, sex, etc.) in step 3 of the regression models. For covariate adjustment, primary models will adjust for age, sex, BMI, race/ethnicity, and smoking status. Secondary models will additionally include physical activity, depressive symptoms, medication use, and socioeconomic status and will be added sequentially. These covariates were selected as they are known to play a role in pain. Finally, a modified Poisson-regression analysis will be performed to assess the relative risk of clinical cognitive impairment between pain impact groups that may have differing nutrition statuses. False Discovery Rate (Benjamini–Hochberg) correction will be applied across primary outcome analyses.

### Study staff training and compliance

Prior to data collection, all study coordinators will undergo standardized training on all study procedures. A detailed checklist for each data collection element is in place to ensure all study coordinators are appropriately trained and able to conduct study procedures. Research coordinators will be certified in each element of the study visit including obtaining informed consent, administering questionnaires, protecting confidentiality of collected data, and performing the test batteries. Retraining of study coordinators will be performed as necessary. All research staff complete human subjects training required by The University of Florida IRB and NIH, including education about the importance of maintaining confidentiality of personal health information. As an additional safety measure, all study staff, graduate students, post-doctoral associates, and senior investigators will be required to maintain active CPR certification.

### Laboratory tests

Participants will have blood drawn at screening and during the post-intervention visit to measure their blood chemistry and will provide urine samples for osmolality tests. Abnormal results will be brought to the principal investigator’s attention immediately. The results will be reviewed and signed by the study clinician. If a participant has both low sodium (<134 mEq/L) and high osmolality (>1,200 L), we will conduct a second blood test. If the sodium is low (<134 mEq/L) in the repeat blood draw, the participant will be excluded as per the UCOPE protocol.

### Assessment of adverse events

A standard operating procedure will be established to collect and deal with adverse events during the research project. Adverse events will be defined as any physical symptoms or side effects that begin following session commencement and will be systematically monitored during the sessions. This will include series of open-ended questions asked by trained research staff regarding any adverse events that have occurred since the last visit or phone contact. Open questions about pain experienced in various parts of the body will be delivered by study staff. Additionally, participants will be asked to complete the WOMAC and PANAS questionnaires to assess pain and feelings. This report will be reviewed by the principal investigator and study physician in a timely fashion. Any serious adverse event, whether related or unrelated to study procedures, will be reported to the IRB immediately.

All reported and observed adverse events will be tracked in a running adverse events participant log, which will contain information regarding dates, description, and severity. All adverse events will be coded using the most current version of the Medical Dictionary for Regulatory Activities (MedDRA).^3^ The number and percentage of participants reporting treatment-emergent adverse events will be tabulated by treatment, system organ class and MedDRA-preferred term. Treatment-emergent adverse events will be those adverse events that begin or worsen following session commencement. Adverse events will be further classified by severity and investigator-assigned relationship to the study variable. All reported and observed adverse events will be tracked on a running adverse events log (specific to each subject and maintained in his/her study file) and will contain information regarding onset/offset dates, time course (i.e., single, sporadic), severity, action taken regarding the event, and whether it meets Food and Drug Administration criteria for “Serious.” Each reported event will be reviewed by the principal investigator and the study physician as soon as possible, within 24 h.

### Data and safety monitoring plan

A Data Safety Monitoring Plan (DSMP) will be in place. Study progress and safety will be reviewed by the principal investigators in collaboration with the study physician. The Independent Safety Monitor (ISM) for this study will be an established senior investigator not associated with this study. A study end report will be compiled and will include a list and summary of adverse events. In addition, this report will address: (1) whether adverse event rates are consistent with pre-study assumptions; (2) reason for dropouts from the study; (3) whether all participants met entry criteria; (4) whether continuation of the study is justified on the basis that additional data are needed to accomplish the stated aims; and (5) conditions whereby the study might be terminated prematurely. The annual report will be signed by the ISM and will be forwarded to the IRB and NIH as a part of continuing reviews. The DSMP will also require that all significant serious adverse events that may be possibly related to study participation will be reported to the IRB and NIH within 48 h of the principal investigators learning of the event. The study will be stopped prior to its completion if: (1) the study is associated with adverse effects that significantly impact the risk–benefit ratio; (2) study recruitment or retention becomes futile; (3) any new information becomes available during the trial that necessitates stopping the study; or (4) if the independent safety monitor indicates that stopping study is necessary due to adverse event frequency or severity.

## Discussion

The aim of this study is to determine the associations among dietary inflammatory potential, epigenetic aging, cognitive status and chronic pain. Previous research shows that chronic pain is characterized by increased chronic immune system activation and neuro-inflammatory processes, such as microglia activation, that may contribute to central sensitization (Vergne-Salle and Bertin, 2021; Ji et al., 2018; Inoue and Tsuda, 2009). ADRDs are also hallmarked by similar neuroinflammatory patterns and characterized by aberrant immune system functioning and subsequent neuroinflammatory processes such as activated microglia within the central nervous system (Heneka et al., 2025). Microglial activation has been found to be present before cognitive decline in ADRD patients, suggesting its early participation in ADRD pathology even before cognitive deficits become apparent (Fan et al., 2017). There is evidence to suggest that such immune dysfunction seen in both chronic pain and ADRDs is regulated by the epigenome, which may serve as a modifiable target of intervention to control the progression of both disease states (Busslinger and Tarakhovsky, 2014). Epigenetic modifiers, such as DNA methylation, histone modification, and microRNA expression, are important modulators of gene expression. These modifiers, influenced by external environmental factors, are what stimulate microglia to transform themselves into their various phenotypes and have been found to be differentially methylated in both patients with chronic pain and patients with ADRDs (Jasiulionis, 2018; Scholz et al., 2024). Epigenetic modifications that lead to immune system dysfunction may be a key player in the overlapping etiologies and pathologies seen in both chronic pain and ADRDs, as they become dysfunctional with aging, and may help explain the potential link between the two conditions (Jasiulionis, 2018). Given the relationship being established between chronic pain and ADRDs, an important question stands out: is chronic pain simply a risk factor for ADRDs, could it be an early symptom, and further, could diet alteration affect both pain and ADRDs?

Diet-induced inflammation and epigenetic alterations may be an important modifiable variable contributing to why individuals with chronic pain are at risk of developing ADRDs. Previously, our group has demonstrated that diet pattern and nutrition status are implicated in chronic immune system activation, systemic inflammation and epigenetic regulation in individuals with chronic pain. The relationship between inflammation and diet pattern is well-documented, as many elements of diet have been reported to activate immune cells and trigger a pro- or anti-inflammatory response. For example, saturated fatty acids in excess have been shown to participate in lipid oxidation, leading to reactive oxygen species and subsequent inflammation (Guerbette et al., 2024). Omega-6 polyunsaturated fatty acids, such as linoleic and arachidonic acid, are precursors for pro-inflammatory prostaglandins, and are consumed in much higher amounts in the typical American diet than anti-inflammatory omega-3 fatty acids (DiNicolantonio and O’Keefe, 2021; Banaszak et al., 2024; Innes and Calder, 2018). Excess carbohydrates increase the presence of Advanced Glycation End Products that can bind to immune cells and stimulate a pro-inflammatory response (Uribarri et al., 2015). In addition to pro-inflammatory effects, a diet can have anti-inflammatory effects as well. A Mediterranean diet can reduce markers of inflammation in individuals with overweight or obesity, in patients with osteoarthritis, and in those with rheumatoid arthritis (Florkowski et al., 2024; Veronese et al., 2024; Hu et al., 2025). Interestingly, one study found that the patients on a Mediterranean diet showed significant reductions in inflammatory markers prior to weight loss, suggesting that the diet itself produced anti-inflammatory effects (Richard et al., 2013). Additionally, evidence from both basic science and clinical studies indicates that diets deficient in various micronutrients, such as Vitamin A and D, are associated with greater pain severity and disability in chronic pain conditions, elevated levels of Aβ and tau proteins in serum and Positron Emission Tomography (PET) imaging, and more severe cognitive impairments in Alzheimer’s disease (Martin and Reid, 2017; Ono and Yamada, 2012). Additionally, as it relates to this study, it has been shown that a Western, pro-inflammatory diet pattern also contributes to pro-inflammatory gene expression (Padin et al., 2019).

This study encompasses a multidisciplinary approach to integrate dietary assessments, epigenetic clocks, cognition, and quantitative sensory testing and represents a major strength of the study. There are also limitations of this work that should be acknowledged. First, the cross-sectional nature of this study does not allow for casual analyses. Mediation and moderation analyses in this study are exploratory. Regarding nutritional assessment, 2 24-h recalls may not fully capture habitual dietary intake, however, the Automated Multiple Pass Method has been shown to improve recall accuracy, and conducting recalls on two separate visits may partially mitigate day-to-day variability. This study also restricted eligibility to those who are considered cognitively intact. Although restricting the sample to cognitively intact individuals improves internal validity for Phase 1, it may reduce large amounts of variability in cognitive outcomes seen in individuals with cognitive decline. Phase-2 of this study plans to include individuals with mild cognitive impairment, and phase 3 plans to include individuals with AD/ADRDs to examine results across healthy cognitive aging and cognitive/diseased states.

## Conclusion

This study is unique in that it explores nutritional neuroscience by integrating diet patterns, the epigenome, pain, and cognition. The present study employs several robust measurements of pain and cognition. Questionnaires assessing pain combined with QST will allow for an all-encompassing assessment of pain impact and processing. This study will also control for pain types, such as high-impact chronic pain, low impact pain, and pain free controls. The impact of pain intensity on risk of cognitive decline will be then explored. The findings of this study may lay the groundwork for dietary mitigation strategies as a strategy to regulate epigenetic aging and prevent chronic pain and ADRDs. In addition to being more cost-effective and lower-risk than most pharmaceutical interventions, nutrition-based interventions carry an extremely positive side-effect profile as they are highly adaptable, can be adjusted to accommodate cost and flavor profiles, and can be tailored to cultural and religious practices, making them a suitable strategy for all regardless of background.

## Footnotes

1
https://pypi.org/project/dii-calculator/

2
dnamage.clockfoundation.org

3
https://www.meddra.org

## Glossary

ADRDs: Alzheimer’s Disease and Related Dementia’s
ANOVA: Analysis of Variance Tests
BDI: The Beck Depression Inventory
BMI: Body Mass Index
BPI-SF: Brief Pain Inventory – Short Form
CMP-14: Comprehensive Metabolic Panel- 14
CPM: Conditioned Pain Modulation
CPT: Cold Pain Threshold
CSQ-R: Coping Strategies Questionnaire-Revised
DII: Dietary Inflammatory Index
DSI: Daily Stress Inventory
DSMP: Data Safety Monitoring Plan
HPT: Heat Pain Threshold
IRB: Institutional Review Board
ISM: Independent Safety Monitor
KOA: Knee Osteoarthritis
MedDRA: Medical Dictionary for Regulatory Activities
MoCA: Montreal Cognitive Assessment
NIH: National Institutes of Health
OA: Osteoarthritis
PANAS: Positive and Negative Affect Scale
PANDA-1: Pain and Nutrition in Dementia and Alzheimer’s Phase 1
PET: Positron Emission Tomography
PPT: Pressure Pain Threshold
PSS-14: Perceived Stress Scale – 14 Item
QST: Quantitative Sensory Testing
SPPB: Short Physical Performance Battery
SWLS: Satisfaction with Life Scale
TICS: Telephone Interview for Cognitive Status
TSMP: Temporal Summation of Mechanical Pain
TSTP: Temporal Summation of Thermal Pain
UCOPE: Understanding Cognition, Oxytocin, and Pain in Elders Study
US: United States
VDS: Verbal Descriptor Scale
WOMAC: Western Ontario and McMaster Universities Osteoarthritis Index
